# Supplementary material for: The role of fibroblast growth factor signalling in Echinococcus multilocularis development and host-parasite interaction
Source: PLoS Negl Trop Dis. 2019 Mar 8;13(3):e0006959. doi: 10.1371/journal.pntd.0006959 (PMC6426264; doi:10.1371/journal.pntd.0006959)
Supplement: S1 Fig — Deduced amino acid sequences of all three E. multilocularis FGF receptors are shown. After the name of each receptor the GenBank accession numbers of the cloned and sequenced cDNAs are shown as well as the gene designation according to the E. multilocularis genome project. Predicted signal peptides are marked in red, predicted IG-domains are marked in blue, tyrosine kinase domains are marked in yellow. Transmembrane regions are underlined. (PDF) [file pntd.0006959.s001.pdf]

EmFR1 (GenBank: LT599044) EmuJ_000833200

MRSGRLLSLVLALLSSASSHQHPPPFSLNGLVFNISTRVAEGAEVCLHCEYFLRPQPKYG 060

FTMIISFLEEASLTRAEVAAAASTQSRFPGNLREAHKKSGRVVNKTIYTPSMRGAKSIFV 120

SKKFFMSSKHAGSYICQMIPVNATSTEGWTLTKWIRLELYHDTVSLKYVWPIFAFGGLPI 180

CLFLVWYCACRPQRMIFKRVLIEKSLLHFAGAPMSSKGHHLAKVTLVPEPRPSLRVYMNA 240

LYQRRGLDHFSEVDEICEVAKKEKEEAKLRVRFLMPSDPTYEVPFGRVQVEGRIGEGAFG 300

LVLRGSAMHLPGGIIGPLPVAIKTLRVNSSDADVVAFVQEIEMMKFIGKHENAIQLYATS 360

NHNGRPVMIMEYAAEGSLVDYLRKNRSWLATQARPMTELVLLCFARQIANGMVYLASKDI 420

VHRDLAARNVVLTKNLVAKIADFGLTRKVEFYYRMKGNGRVPLKWMAPESVFQKVFTSKS 480

DIWSFGVLLWELFSLGDSPAANLSVNEFLEALHLGPSLYTKPKYADEEVFSGLMQPCWEH 540

QPECRPTFTDILATLSKFLQGC 562

EmFR2 (GenBank: LT599045) EmuJ_000196300, EmuJ_000196200

MCLRALCLTALFLVKFCHVIAHPVYYVSTGEKAVLDFIFSHSSKTSAAVVQWTFLPFLSSI 060

PEGENVENGFHLNTSYHSSRYKNDISQVICPESFIGPPGRTCRLARLSISQVIPADEGIY 120

RARIVGTNTTTADCYPCTGELKLRVLVDENDVGQPMSGDHSDGLMSSEDFMGSNDIPIYF 180

YAPPFSLDFNMNSTVCVGQNFEFQCRAEPINYGINLTLYRLDPLFTLSSLEDANNHGTGI 240

DFRNTNTRVIQTSSGRKAKSRHLKYKLHSVSMNDTGGYLCVASVPNMHAARWSYMFLQVE 300

DCASLSALASTMPFRERTLEVFTSPSALTFICLLLAILLVTLTALVFLAWRQQRLPLFLF 360

GSKKWSKAANKVVREANLLYPWSAPPFHNCSHQVAKDATNASTASYVGIRSDGACSGVGR 420

NLQVPAVIIQVDTSVTAGGDGGGGGENGGEAEGKLSAYKIPSDPAWEVPRDCIRLGRQIG 480

AGAFGVVFAGVISEPAKVLPGLRRGDPLDRPLCSAQELTVAVKTLRNNFTEQELADLVRE 540

MEILKQFEPHPHVIQFYGACTQNGRLQMLVELAPYGNLRDFLMDRRPRAGHKVINQPLSR 600

LYTRHLVSFGLQVARGMDYLTRLNIVHRDLAARNILIGNRFVAKIADFGLTRAVCDYYKK 660

CSDGRLPIKWMAPESIFDRRYTTKSDVWSFGILLWEIFSFGGTPYPTLSAEALLKALQLG 720

FRNEQPLASPPSVYNLMLACWSIDPVARPSFAHLVSSLQQVYDDTSASSSISSASSSSLS 780

STSSSSSSRSSSPTVSTVYLTLNNTITNVNTDSNNGGLHNYNNQGVHTSTVSAPISPSIY 840

ESTAPRLPLGEQCPSSSLSGNFQGHQGPLGPPAGGGNSVLVNNCYAEQGQYLQSIHKAFS 900

MHCNYVVPPRSSE 913

EmFR3 (GenBank: LT599046) EmuJ_000893600

```
MVLIPQMLPVILLHVAAVGCVRIEVPVFTDYFMPCNISALPARPRMEDFYWFQDGRLNRG   060
VNDFLLPQGEIPFEGISPGAAGAYRCCYNGSTKITCAEVVNLIVKAPRPNIFQHTLLNIT   120
PYIFPNNVYWLPQMLTTPLEVEMTTEPLEFNCFYFAQSSATRRPHVAWYFNDLYPEDRRF   180
ESPIQEDDKRYVVKTMEITCEEDYMQHSRSRCFKSTLMVRLPSDLRRRTVYTCEVQMFKA   240
GGLVENQLSVDYRLKSDNRRDSDWIYGLTDIERFGEDCDRFPNELPPLEALIQELNACKR   300
NSFLRLWVSPANSEKLVQATCSRPVFLVFDELLELIRLPPDPHVIRLFFKVENITSVRSP   360
FDGILKRYGKFLLKSNVTTLRVGGKKHHSFDTTLQGNVSLDCQPSHVVVCVYGPLVQMKL   420
VHMACSPSDRHEGFLLICMVVGGLVVATLLFAGLYLWRRQCLSRRHYAVWKTVEAYTSSL   480
LLERRLCSPPLPTPLPRGPNNFHREAAQAKKVAVLEYVKLGKTRWPLSARSLRLEEQIAC   540
GSYGDVFKGVLLTSPPGQSVLPRPIVAKVLNDVYLKDHVLEFANEVAILRLIGAHPTIIQ   600
FLGCAHRTNLSNRPVLVTEYASHGTLLGYLRALRPNRDTALGEVVMTYWWTRARTLVADL   660
YSFVIDIASALVYLEEIAVVHSDVAARNVLLTASLTAKLNDFGLACVIPHGKFVELPATK   720
KVPVRWSAPEVLQENRRHARSDVWSFGVLLWETFAVGETPYADLPSESAVGVFVGREGGR   780
LPRPTLASGTLYSLMTACWASSVNARPDFRTLLEDLSREAALERKKSEDANLLSHVGEDL   840
LFTPVVEAPIKRSF                                                 854
```
