# Supplementary figures and images for: The role of fibroblast growth factor signalling in Echinococcus multilocularis development and host-parasite interaction
Source: PLoS Negl Trop Dis. 2019 Mar 8;13(3):e0006959. doi: 10.1371/journal.pntd.0006959 (PMC6426264; doi:10.1371/journal.pntd.0006959)

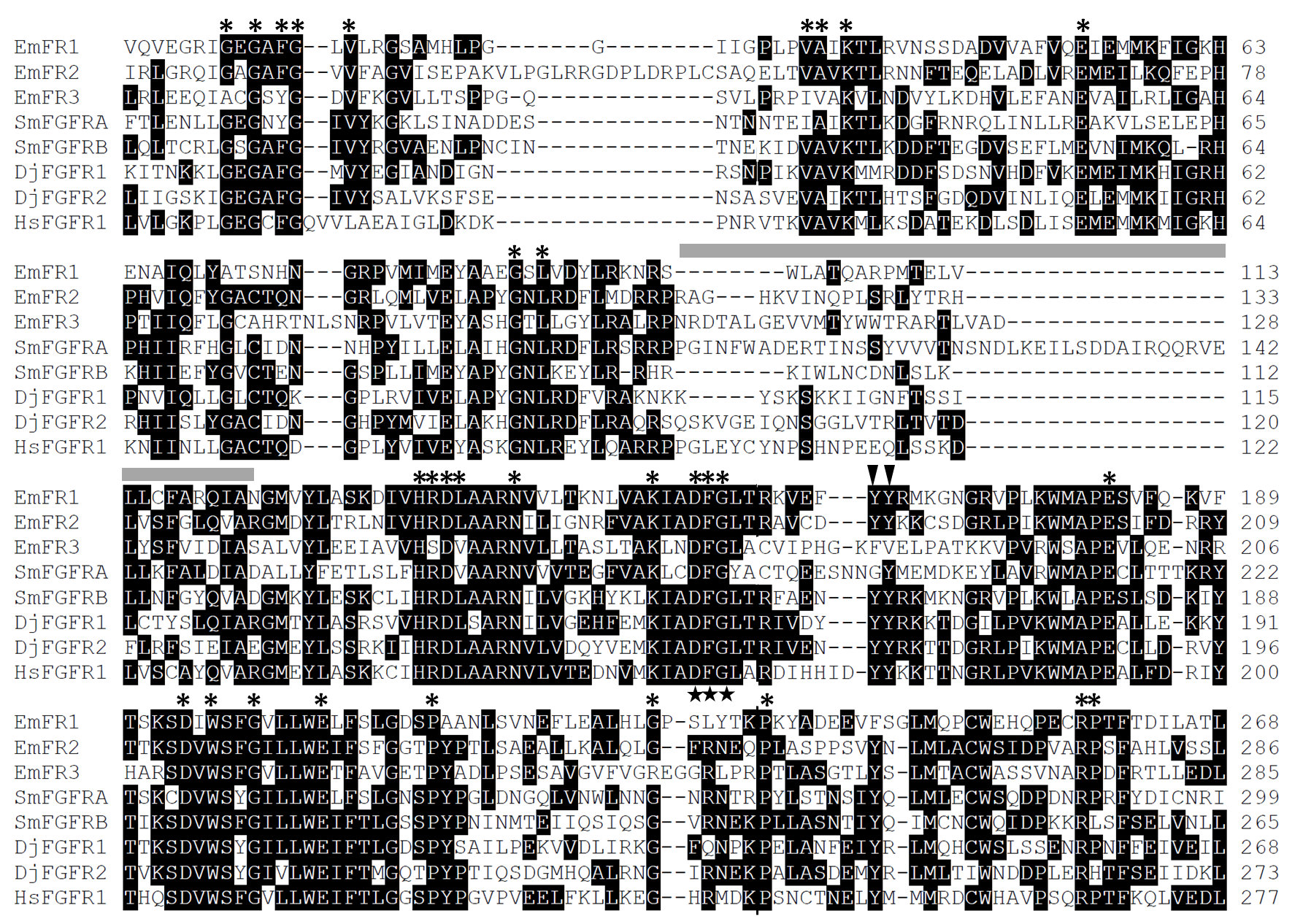

Supplement: S2 Fig — Depicted is a CLUSTAL W amino acid sequence alignment of the TK domains of E. multilocularis EmFR1 (GenBank accession no. LT599044), EmFR2 (LT599045) and EmFR3 (LT599046) with those of Schistosoma mansoni FGFRA (SmFGFRA; Wormbase accession number: Smp_175590.1) and FGFRB (SmFGFRB; Smp_157300.1), Dugesia japonica FGFR1 (DjFGFR1; NCBI accession no.: Q8MY86) and FGFR2 (DjFGFR2; BAB92086.1), and the human FGFR1 receptor (HsFGFR1; NP 075598.2). Amino acid residues that are identical to the consensus of all sequences are printed in white on black background. Numbering to the right starts with amino acid 1 of the TK domain. The insert region of the split TK domain is indicated by a grey bar. Amino acids known to be highly conserved among tyrosine and serine/threonine kinases [62] are indicated by asterisks above the alignment. The two tyrosine residues known to be important for full activation of the human FGF receptor [58] are marked by black triangles. The TKD DFG motif which was modified to DNA to generate kinase-dead FGF receptors is indicated by black stars below the sequence. (TIF) [file pntd.0006959.s002.tif]

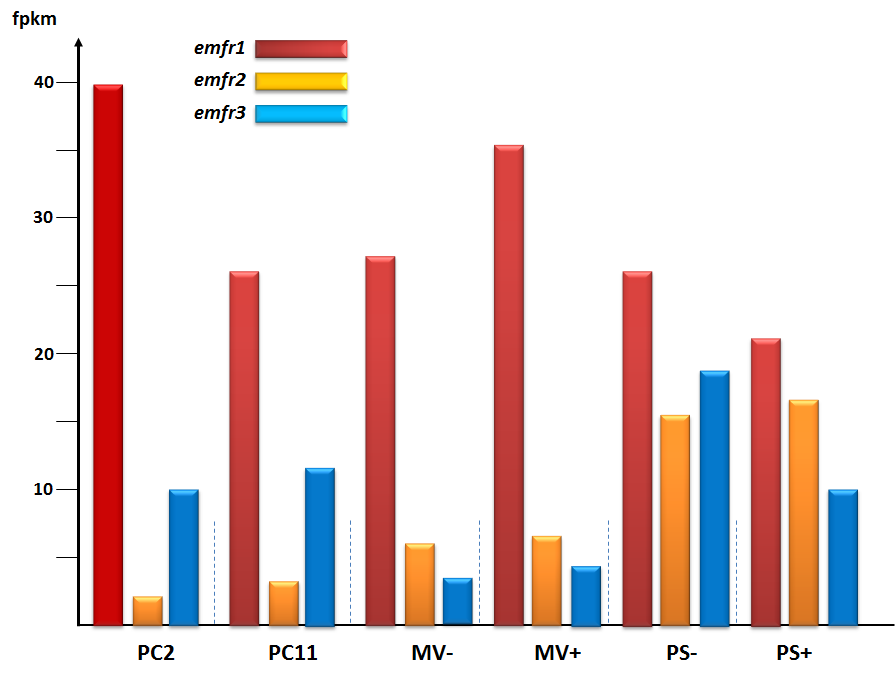

Supplement: S3 Fig — Indicated are fpkm (fragments per kilobase of transcript per million mapped reads) values for emfr1 (red), emfr2 (orange), and emfr3 (blue) in E. multilocularis primary cell cultures after 2 days of incubation (PC2), after 11 days of culture (PC11), in mature metacestode vesicles without (MV-) and with (MV+) brood capsules as well as in dormant (PS-) and activated (PS+) protoscoleces. Transcriptome data have been generated during the E. multilocularis genome project and were mapped to the genome as described in [14]. (TIF) [file pntd.0006959.s003.tif]

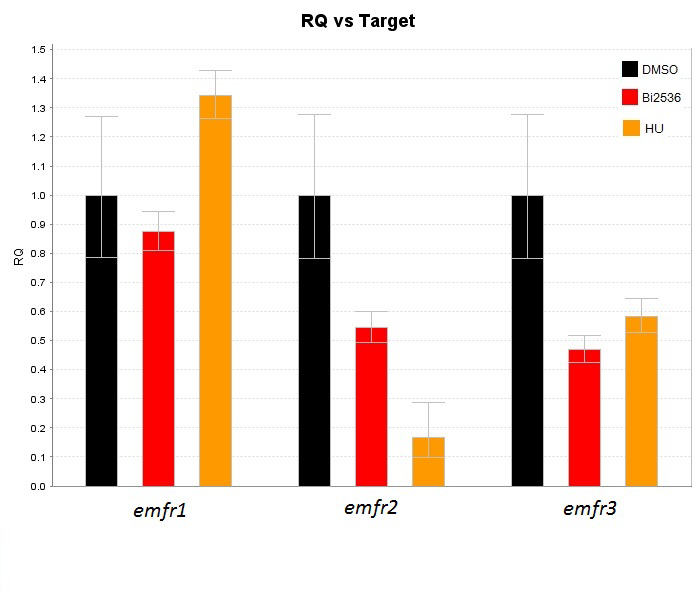

Supplement: S4 Fig — Metacestode vesicles cultivated for 7 days in the presence of DMSO (control, black) or in the presence of hydroxyurea (HU, yellow) or BI 2536 (BI, red) were used for RNA isolation and cDNA preparation. Expression levels of emfr1, emfr2, and emfr3 (as indicated) were measured using the constitutively expressed gene elp as a normalization control (set to 1.0). All experiments were carried out in triplicates. (TIF) [file pntd.0006959.s004.tif]

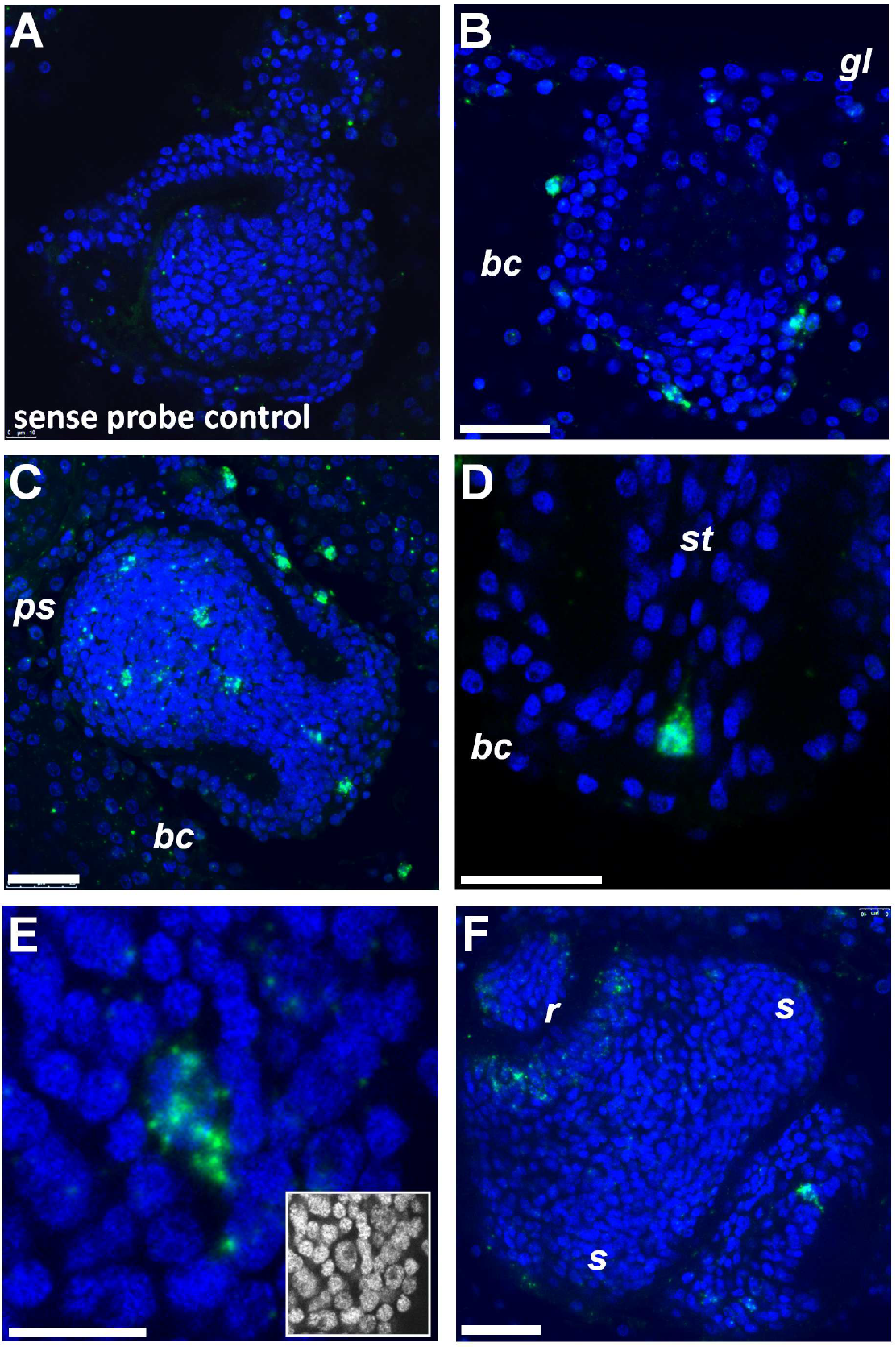

Supplement: S5 Fig — In all panels the WMISH signal is shown in green, and DAPI nuclear staining is shown in blue. A. Sense probe (negative control). B. Early brood capsule formation. C. Early protoscolex formation. D. Detail of early protoscolex formation, showing an emfr3+ cell in the region of the stalk connecting the developing protoscolex to the brood capsule. E. Detail of early protoscolex formation, showing the morphology of an emfr3+ cell (inset: only DAPI channel is shown). F. Late protoscolex formation. bc, brood capsule; gl, germinative layer: ps, protoscolex; r, rostellum; s, sucker; st, stalk. Bars: 25 μm for B, C, D and F; 10 μm for E. (TIF) [file pntd.0006959.s005.tif]

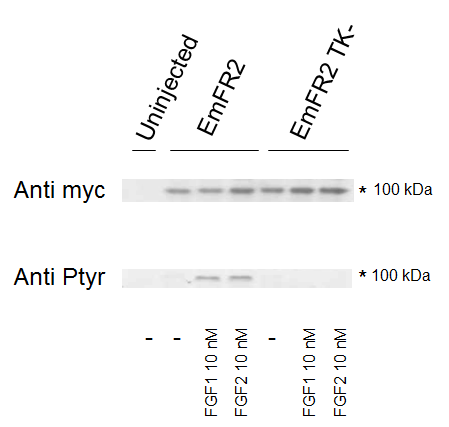

Supplement: S6 Fig — E. multilocularis EmFR2 (EmFR2) and a kinase-dead version of EmFR2 (EmFR2 TK-) were expressed in Xenopus oocytes and stimulated with either 10 nM FGF1 or 10 nM FGF2 as indicated. After stimulation, cell lysates were generated, separated by SDS-PAGE and analysed by Western blot using antibodies against the myc-tag (Anti-myc; loading control) or phosphorylated tyrosine (Anti Ptyr). Depicted are the results for EmFR2. Results for EmFR1 and EmFR3 were comparable. (TIF) [file pntd.0006959.s006.tif]
